# Prevalence of positivity in patch tests and reactivity to substances present in the main dressings in patients with chronic leg ulcers^☆^

**DOI:** 10.1016/j.abd.2024.05.003

**Published:** 2024-11-09

**Authors:** Ísis Fiorello de Oliveira Mesquita, Larissa Pierri Carvalho Fonseca, Maria Rita Parise Fortes, Hélio Amante Miot, Luciana Patricia Fernandes Abbade

**Affiliations:** a Check-up Sector, Hospital Sírio Libanês, São Paulo, SP, Brazil; b Postgraduate Course in Cosmiatry and Laser, Instituto Lapidare, Balneário Camboriú, SC, Brazil; c Department of Infectology, Dermatology, Imaging Diagnosis and Radiotherapy, Faculty of Medicine, Universidade Estadual Paulista, Botucatu, SP, Brazil

Dear Editor,

Chronic lower limb ulcers (CLLUs) are lesions that occur below the knee and last for more than six weeks. They are usually venous ulcers (60%–70%), arterial or mixed ulcers (10%–25%), and neuropathic ulcers, and affect 1%–7% of the population over 65 years of age, representing an important public health problem.^1^

In CLLUs, allergic contact dermatitis (ACD) occurs in approximately 50% of patients, and can impair healing.^2^ ACD occurs due to repeated contact with low molecular weight allergens, breakdown of the skin barrier, and particularly due to prolonged use of topical therapies for the treatment of CLLUs.^3^ Patch testing is indicated in patients with peri-ulcer eczema.

The most prevalent allergens vary according to the geographic region, popular practices, and genetic characteristics of each population. Since there are few studies on the subject and, to date, none in Brazil, this study aimed to identify the prevalence of positivity to patch tests and reactivity to substances present in the main dressings used in patients with CLLUs.

A cross-sectional study was conducted in patients with CLLUs treated at the chronic ulcers outpatient clinic of the Dermatology Service of Hospital das Clínicas, Medical School (FMB), São Paulo State University (Unesp), Botucatu Campus. The project was approved by the local Research Ethics Committee (CAAE 47938715.9.0000.5411) and the selected participants signed the free and informed consent form. The inclusion criterion comprised a diagnosis of CLLU, regardless of the etiology. The exclusion criteria comprised the use of systemic immunosuppressants in the last thirty days; the presence of dermatoses that prevented the performance of the patch test on the back region; and pregnancy.

The patch test was performed using a standardized battery for the Brazilian population (FDA Allergenic, Rio de Janeiro, Brazil), which consists of 30 substances fixed in Finn Chambers®. It was also performed with the twelve main dressings and topical products used for CLLUs and peri-ulcer skin: essential fatty acid oil, silver sulfadiazine, collagenase with chloramphenicol, mupirocin, hydrogels, hydrocolloids, elastic compression bandage, Unna’s boot, hydrofiber with silver, silver activated charcoal, calcium alginate, and micropore. Readings were performed at 48 h and 72 h.

A total of 78 participants with CLLUs were included, with a predominance of females (47/78−60.9%) and a mean age (standard deviation) of 68.4 (12.5) years. The etiology of the ulcers was venous in 67/78 (85.9%), mixed (venous and arterial) in 9/78 (11.5%), and arterial in one participant (1.3%). The median (p25-p75) duration of the ulcers was 24 (10–120) months. Peri-ulcer eczema was present in 56/78 participants (71.8%; Fig. 1). There was a personal history of peri-ulcer eczema in 27/78 (34.5%) participants; 17/78 (21.8%) had a history of eczema in other sites besides the peri-ulcer region and 11/78 (14.1%) had a history of atopy. Table 1 shows the main products and dressings that were reported as having been used on the ulcer and peri-ulcer region.

**Fig. 1:**
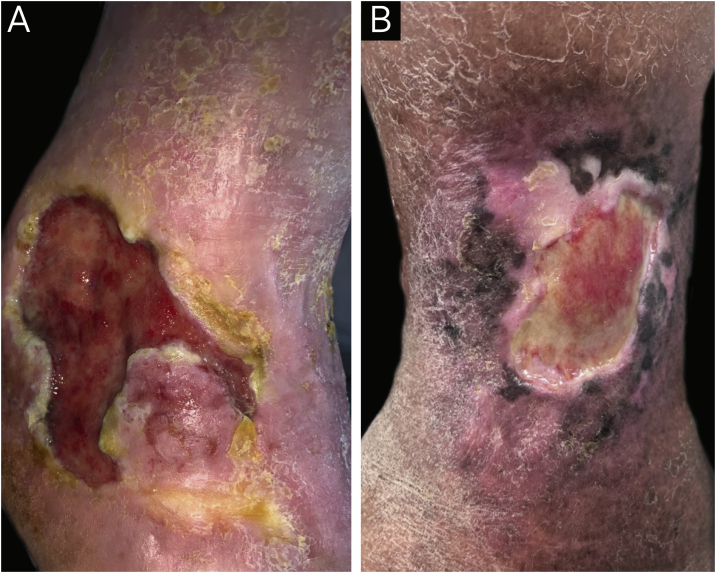
(A) Patient with venous ulcer and peri-ulcer eczema characterized by pruritus, erythema and desquamation. The patch test showed a positive reading at 72 hours, with relevance for lanolin, paraben mix, hydrogel, silver sulfadiazine, elastic bandage, Unna’s boot, collagenase with chloramphenicol and hydrofiber with silver. (B) Patient with venous ulcer and peri-ulcer eczema. The patch test did not show a positive result with the standard battery; however, the patch test with dressings showed a positive test at 72 hours for hydrogel and collagenase with chloramphenicol.

**Table 1:** Compression therapy, dressings and products that were reported as previously used by the 78 participants in the ulcer and peri-ulcer region.

Variable	Value
**Compression therapy**^a^, n (%)	
Elastic bandage	65 (83.3)
Elastic stockings	44 (56.4)
Unna's boot	34 (43.6)
Did not use	06 (7.7)
**Topical antibiotic**^a^, n (%)	
Did not use	35 (44.9)
Silver sulfadiazine	32 (41.0)
Neomycin/Nebacetin	22 (28.2)
Mupirocin	8 (10.3)
Fusidic acid	1 (1.3)
Gentamicin	5 (6.4)
**Essential fatty acid, n (%)**	47 (60.3)
**Fibrolysin with chloramphenicol, n (%)**	40 (51.3)
**Collagenase with chloramphenicol, n (%)**	54 (69.2)
**Peri-ulcer topical corticosteroid**^a^**, n (%)**	
Did not use	39 (50.0)
Betamethasone	29 (37.2)
Dexamethasone	19 (24.4)
Betamethasone + Gentamicin	7 (9.0)
**Occlusive dressings**^a^, n (%)	
Hydrogel	62 (79.5)
Silver activated charcoal	46 (59.0)
Hydrofiber with silver	40 (51.3.)
Hydrocolloid	35 (44.9)
Calcium alginate	25 (32.1)
Polyurethane film	4 (5.1)
**Peri-ulcer skin moisturizer, n (%)**	42 (53.8)

a The same participant may have used more than one item.

Regarding the contact test with the standard battery (Table 2), it was positive in 72 h in 31/78 (39.7%, 95% CI 30%–49%) participants, and the main allergens were: paraben mix (9/78‒11.5%), nickel sulfate (5/78‒6.4%) and lanolin (3/78–3.8%). Some substances that were positive are not relevant to patients with peri-ulcer eczema because they are not related to the dressings and products usually used by these patients.

**Table 2:** Positive patch test (+ to +++) with the standard battery after 72 hours and its relevance in relation to peri-ulcer eczema, in 78 participants.

Substance	Positive test at 72 h reading^a^, n (%)	Relevance in relation to peri-ulcer eczema
Paraben mix	9 (11.5)	Yes
Nickel sulfate	5 (6.4)	Yes
Lanoline	3 (3.8)	Yes
Balsam of Peru	2 (2.6)	No
Kathon CG	2 (2.6)	Yes
Thimerosal	2 (2.6)	Yes
Colophonium	2 (2.6)	Yes
Formaldehyde	2 (2.6)	No
Propylene glycol	1 (1.3)	Yes
Cobalt chloride	1 (1.3)	Yes
Thiuram mix	1 (1.3)	No
Ethylenediamine	1 (1.3)	No
Quinoline	1 (1.3)	No

a The following substances from the standard battery did not show positivity in any patient within 72 hours: potassium dichromate, neomycin, perfume mix, quaternium-15, nitrofurazone, turpentine, carba mix, paraphenylenediamine, anthraquinone, PPD, hydroquinone, para-tertiary butylphenol, Irgasan, Mercapto mix, epoxy resin, benzocaine and promethazine.

In the tests with dressing substances (Table 3), 22/78 (28.2%; 95% CI 18%–37%) participants showed reactivity to these components within 72 h. The main allergens were: collagenase with chloramphenicol (13/78‒16.7%), silver sulfadiazine (11/78–14.1%), and hydrogel (6/78–7.7%). Another relevant aspect was that 5/78 (6.4%) participants showed a reaction to micropore; however, with a positive reading only in the first 48 h, then negative in the 72 h reading, demonstrating to be an irritant reaction and not ACD.

**Table 3:** Positive patch tests at 72 hour readings (+ to +++) for products and dressings used in 78 participants.

Substance	Positive test at 72 h reading^a^, n (%)
Collagenase with chloramphenicol	13 (16.7)
Sulfadiazine with silver	11 (14.1)
Hydrogel	6 (7.7)
Unna’s boot	4 (5.1)
Essential fatty acids	3 (3.8)
Elastic bandage	2 (2.6)
Hydrofiber with silver	2 (2.6)
Mupirocin	1 (1.3)
Silver activated charcoal	1 (1.3)

a The following substances in the patch tests with dressings did not show positivity in any patient at 72 hours: hydrocolloid, calcium alginate and micropore.

When the results of the patch test were analyzed for the standard battery and for the dressings concomitantly, 39/78 (50%; 95% CI 39%–59%) of the participants reacted to some of the tested substances, and 14/78 (17.9%; 95% CI 10%–24%) participants reacted to both the standard battery and the dressings (Fig. 1A).

A high percentage of positive allergy patch tests was observed, in agreement with recent literature.^3^ However, the positivity was lower than that of older studies, which varied between 75% and 80%, leading to the hypothesis that substances known to be more allergenic have been avoided in the composition of dressings and products for treating ulcers.^4^, ^5^

The substances in the standard patch test that showed the most reactivity in the present study were paraben mix, nickel sulfate and lanolin. Other allergens that showed reactivity in a few participants in the present study, but were highly significant in other samples were neomycin, perfume mix, carba mix, benzocaine and hydroquinone.^5^

Paraben mix is a preservative used in the cosmetic, pharmaceutical and food industries. It is reported that patients with CLLUs may react to parabens used in cosmetics applied to the peri-ulcer skin but tolerate them on healthy skin.^6^ Regarding lanolin, which is used in dermocosmetics such as creams and shampoos, the present study showed positivity similar to that reported in other studies. Nickel sulfate has shown low positivity in other studies, but had high positivity in the present study (12.8%). In the general population with suspected ACD, the prevalence is approximately 36%.^7^

In tests with dressing substances, the present study identified 28.2% with at least one positive test, a result similar to that found in other series.^3^, ^5^ This demonstrates the importance of performing patch tests with specific dressing substances, in addition to the standard battery.

The most reactive dressing substances in this sample were: collagenase with chloramphenicol, silver sulfadiazine and hydrogel. Collagenase contains a proteolytic enzyme capable of digesting collagen and is widely used for debridement of ulcers. However, it is difficult to differentiate whether the reactivity is due to clostridiopeptidase A or chloramphenicol.^8^ Hydrogels and hydrocolloids are mentioned as allergens, with positivity in other studies in up to 23% of patients tested for hydrogel and 52% of those tested for hydrocolloids.^3^, ^9^ Sensitization to hydrogels seems to be related to propylene glycol, and sensitization to hydrocolloids may be due to carboxymethyl cellulose and colophonium derivatives.^10^

The limitations of the present study were due to the majority inclusion of venous ulcers, with little representation of other causes of CLLUs. The authors did not test all products and dressings available in the national market, but rather the main products used in the treatment of CLLUs, and collagenase was not tested by itself. Moreover, control tests were not performed with the dressings, and since they were of certain commercial brands, it was difficult to identify the actual allergen.

In conclusion, there was a high positivity in the patch tests in patients with CLLUs, with emphasis on paraben mix, nickel sulfate, lanolin, collagenase with chloramphenicol, silver sulfadiazine, and hydrogel. These results highlight the relevance of performing patch tests with a standard battery and with the dressings/products usually used in patients with peri-ulcer eczema.

## Authors' contributions

Ísis Fiorello de Oliveira Mesquita: Design and planning of the study; collection, analysis and interpretation of data; drafting and editing of the manuscript; critical review of the literature; critical review of the manuscript; approval of the final version of the manuscript.

Larissa Pierri Carvalho Fonseca: Design and planning of the study; collection, analysis and interpretation of data; drafting and editing of the manuscript; critical review of the literature; critical review of the manuscript; approval of the final version of the manuscript.

Maria Rita Parise Fortes: Collection, analysis and interpretation of data; drafting and editing of the manuscript; critical review of the literature; critical review of the manuscript; approval of the final version of the manuscript.

Hélio Amante Miot: Analysis and interpretation of data; statistical analysis; drafting and editing of the manuscript; critical review of the literature; critical review of the manuscript; approval of the final version of the manuscript.

Luciana Patricia Fernandes Abbade: Design and planning of the study; analysis and interpretation of data; drafting and editing of the manuscript; critical review of the literature; critical review of the manuscript; approval of the final version of the manuscript.

## Financial support

None declared.

## Conflicts of interest

None declared
